# Qualitative Aspects of Some Traditional Landraces of the Tomato “Piennolo” (*Solanum lycopersicum* L.) of the Campania Region, Southern Italy

**DOI:** 10.3390/antiox9070565

**Published:** 2020-06-28

**Authors:** Florinda Fratianni, Autilia Cozzolino, Antonio d’Acierno, Filomena Nazzaro, Riccardo Riccardi, Patrizia Spigno

**Affiliations:** 1 Institute of Food Science, CNR-ISA, Via Roma 64, 83100 Avellino, Italy; florinda.fratianni@isa.cnr.it (F.F.); dacierno@isa.cnr.it (A.d.); 2 Department of Agricultural, Environmental and Food Sciences (DiAAA)-University of Molise, Via de Sanctis snc, 86100 Campobasso, Italy; autilia.cozzolino@unimol.it; 3 Cooperativa ARCA 2010, 80011 Acerra, Italy; ricc.riccardi@libero.it (R.R.); patspigno@hotmail.com (P.S.)

**Keywords:** tomato “Piennolo”, biodiversity, chemical composition, antioxidants, polyphenols, lycopene, ascorbic acid

## Abstract

Our study aimed to analyze some qualitative aspects of five landraces of the tomato “Piennolo,” typical of the Vesuvian area, Italy, and determine the in vitro antioxidant activity. All samples showed a high °Brix value and acidity, as well as a discrete amount of reducing sugars, indicating their good quality as fresh products. They showed a high content of lycopene (up to 218 μg g^−1^ of fresh product) and ascorbic acid (up to 238 μg g^−1^ of fresh product). The content of total polyphenols was never less than 278 μg g^−1^ of fresh product. Hyperoside, chlorogenic and gallic acids were the most abundant polyphenols. The interrelationships between the parameters analyzed and the different landraces showed that total polyphenols could have mostly affected (ρ = 0.76) the antioxidant activity more than lycopene (ρ = −0.96). The interrelationships between the most abundant polyphenols and antioxidant activity showed that hyperoside, although the most abundant, negatively affected (ρ = −0.93) the antioxidant activity. Due to the high content of lycopene, ascorbic acid, and hyperoside, the five landraces of the tomato “Piennolo” could be considered promising in terms of their potential healthy characteristics.

## 1. Introduction

The current needs of agriculture and agro-industry have induced farms to pursue certain strategies with the aim of increasing productivity and reducing the costs of production to maintain an adequate level of competitiveness [1], as they are becoming overwhelmed in an increasingly globalized economic scenario. Consequently, genetic erosion involving the traditional landraces with specific and generally high organoleptic uniqueness (due to the selection made by farmers over the decades and even over the centuries), were gradually subjected to a disappearance or a decrease, with the consequent standardization of production and a flattening of the qualitative and organoleptic aspects. In this manner, we have witnessed the loss of the traditional union between the genotype and cultivation environment, a decrease of genetic resources and the concurrent selection of the raw material based almost exclusively on its suitability for industrial transformation. Therefore, the industrial derivatives are characterized by a certain homologation of “taste” and the impoverishment of the traditional link between territory, traditions, and food habits. [2,3]. However, in recent years, we have observed an opposite trend by consumers, who have started to establish a different orientation in their choice of food products, with the aim of recovering the traditional gastronomic heritage of their own territory [4]. The Campania region, located in Southern Italy, is an important source of local and valuable local varieties [5,6]. Small, traditional family farms play an important role in conserving a large genetic heritage, representing a concrete example of safe-guarding biodiversity [7,8]. The tomato fruit (*Solanum lycopersicum*) constitutes one of the most popular products cultivated worldwide [9] and provides important dietary nutrients and antioxidants, such as lycopene, carotenoids, phenols, and flavonoids, all of which are important elements for the human diet [10]. Concurrently, such biomolecules represent a mode of action exhibited by the plant to counteract the effects of parasites and insects, and have antimicrobial activity [11]. The tomato has been a symbolic crop for Italian agriculture [12,13]. In the Campania region, as with other parts of traditional agriculture, the tomato sector also experienced a gradual down-sizing at the end of the 1980s, which has slowed down in recent years due also to the competition with more powerful agro-industrial companies and to the depletion of the traditional genetic varieties. Therefore, Campania region is striving to further enhance this sector and safeguard the indigenous varieties present in its territory, going to identify, both through the financing of projects and the action of the so-called “custodian farmers”, the most susceptible varieties of enhancement, which can give further economic and social development to agriculture [13]. Although Campania is famous above all for the cultivation of the typical tomato used for the preparation of canned and peeled tomatoes, such as the “San Marzano” tomato, recovered after decades of neglect and widely studied [14], several different landraces of tomato are present and fortunately still cultivated and preserved in the regional territory. These include the “Pomodorino del Piennolo”, one of the oldest and most typical products of regional agriculture, which is prized to the extent that it is also represented in the scene of the traditional Neapolitan nativity. In Campania, there are numerous landraces of the tomato “Pomodorino del Piennolo”, most commonly called “Piennolo” with its small size, organoleptic quality, typicality, and rusticity [15,16]. The most famous ecotypes are those cultivated in the area of the Vesuvius volcano and constitute a grouping of old local cultivars and biotypes linked by more or less similar morphological and qualitative features, whose selection has been handled over the decades by the farmers themselves, who gave them characteristic names such as “Fiaschella”, “Lampadina”, “Patanara”, “Principe Borghese”, and “Re Umberto”. The “Piennolo” tomato has an oval shape with a pointed apex. The peel is thick, and the size does not exceed 25 g. The pulp of the “Piennolo” tomato has a high consistency, an intense red color, and a lively, intense and sweet-sour taste. The tomato “Piennolo” is stored using still the ancient practice “at piennolo” that is, bunches of small tomatoes are tied together—the so-called “scocche”—to form a large bunch (with an overall weight of 1–1.5 kg) and suspended in a well-aerated location, which can be used until the end of winter. Therefore, in the Mediterranean area, different varieties of tomato show similar peculiarities, such as the so-called tomato “de penjar” [17]. “Piennolo” tomato, like other traditional varieties of tomato distributed in the Mediterranean area, belong to the so-called “long storage (LS)” tomatoes that, as described by Conesa et al. [3], do not need postharvest ripening nor cold temperature storage; in addition, they are drought tolerant enough, due to the typical open field cultivation under semi-arid Mediterranean summer conditions, meaning that plants are irrigated only during first stages after transplantation and rely on occasional rain-fed afterwards in contrast with the commercial practice for most tomato cultivars. LS tomatoes were diffused several centuries ago, in many regions of the Mediterranean area, from the Eastern Iberian Peninsula, to the Balearic Islands, in the Southern Italy and Sicily; all these regions were part of the Crown of Aragón from the end of 13th to early 18th centuries [18]. Therefore, some of LS tomatoes, including “Piennolo”, were recently the focus of research in the H2020 project TRADITOM (N. 634561), focused on the identification and valorization of European traditional tomato varieties and their cultural practices. At national level, Campania region, through two main projects, “SALVE” and “AGRIGENET”, supported the characterization of several long storage and not long storage autochthonous landraces of tomato, including “Piennolo”. The peculiarities of the tomato “Piennolo” are the high consistency of the peel, the attachment strength to the peduncle and the high concentration of sugars, acids and other soluble solids, which make it a long-life product during which none of its organoleptic qualities undergo alterations. Such particularities are deeply linked to the soil and the climatic factors typical of the geographical area in which the tomato is grown, where the soils, of volcanic origin, are made up of pyroclastic material originating from the eruptive events of the Somma-Vesuvius volcanic complex. Carillo et al. [15] reported the sensory and qualitative analysis of different “Piennolo” ecotypes, such as “Acampora”, “Cozzolino”, “Riccia di San Vito”, “Fofò”, “Lucariello”, and “Zeno”, which are also typical of the Vesuvian volcano area. Caruso et al. [19] evaluated the nutritional quality of the Vesuvian Piennolo tomatoes related to the farming systems, and characterized them in terms of the content of some minerals, such as K, P, Mg, and for their polyphenol content, lycopene, glutamate, gamma amino butyric acid (GABA), and glutamine. Our study aimed mainly to (i) analyze some parameters such as the °Brix, pH value, acidity, and reducing sugar content of five landraces of the tomato Piennolo, grown in an unique area near Naples; (ii) evaluate the content of β-carotene, lycopene, total polyphenols, phenolic profiles, ascorbic acid, and polyphenol profile; (iii) determine the in vitro antioxidant activity through the 2,2-diphenyl-1-picrylhydrazyl (DPPH) radical-scavenging activity; and (iv) attempt to identify, through the analysis of correlation, the factor—or factors—which could affect more or less the antioxidant activity.

## 2. Materials and Methods

### 2.1. Chemicals

Caffeic acid, ferulic acid, *p*-coumaric acid, gallic acid, chlorogenic acid, catechin, epicatechin, hyperoside, rutin, quercetin, 2,2-diphenyl-1-picrylhydrazyl (DPPH), β-carotene, ascorbic acid, HPLC-grade methanol, NaOH, sulphuric, metaphosphoric, acetic and formic acids, acetonitrile, petroleum ether, ethanol and acetone were purchased from Sigma-Aldrich (Milano, Italy). Apigenin was purchased by Extrasynthese (Genay, France). The Folin–Ciocalteu reagent was purchased by BIO-RAD (Milano, Italy). Water was distilled and filtered through a Milli-Q apparatus (Millipore, Milano, Italy) before use.

### 2.2. Plant Material

Five landraces of the tomato “Piennolo”, listed by the Official Bulletin of the Campania Region (B.U.R.C. n°42, 145, 2009), were all grown and collected in the farm of the “Cooperativa ARCA 2010” sited in Acerra (NA), Italy. Acerra is characterized by a Mediterranean climate with an average of air temperature (T), humidity (U), and rainy days (R) = 22.7 °C; U = 63.8%; R = 6.6 during the growing season [20]. The seeds were deposited in a gene bank (“banca del germoplasma” of the Campania Region) and stored at constant temperature (4 °C) and humidity (60%). Each landrace was cultivated in an area of 72 m^2^. The irrigation and plant protection as well as the weed control were carried out following local practices. For the experimental design we considered three replicates and 10 plants per replicate. Tomato fruits were harvested based on the same sun exposition, mature ripe stage (according to the full appearance of red color on the fruit surface) and size, and gently cleaned. Peduncles and seeds were removed, some of the fresh fruit was subjected to chemical analysis, and other samples were instantly stored at −30 °C for the biochemical analysis.

### 2.3. Chemical Analysis

Frozen diced tomato sample was quickly thawed by placing under running tap water. They were homogenized (Stomacher^®^ 400 Circulator, VWR International Srl, Milano, Italy) for 3 cycles × 1 min and filtered through Whatman N.l filter paper (Whatman International Ltd., Maidstone, UK). The residue was discarded, while the filtrate was used for chemical analyses. The refractometric index (°Brix), pH value, titratable acidity, and amount of reducing sugars were determined following the Italian National Official bulletin (1989) for the analysis of vegetable and vegetable-derived products [21]. Refractometric index (°Brix) was determined by a HHTEC refractometer (GMBH, Heidelberg, Germany); pH values were determined by pH meter (Hanna, Metter Toledo, Milano, Italy); titratable acidity was determined by potentiometric titration with NaOH 0.1 M pH 8.1, using 10 mL of juice, and was expressed as % of citric acid; determination of total reducing sugars was assayed with the Fehling solution, using methylene blue as indicator.

#### 2.3.1. Total Polyphenols

Samples were homogenized (Stomacher^®^ 400 Circulator, VWR International Srl, Milano, Italy) and incubated (1:3 w/vol) in methanol (containing acetic acid 1%) overnight at 4 °C. After centrifugation (11,600× *g*, 15 min; Biofuge, Beckman Italia), supernatants were recovered; pellet was treated again with the same extractive solution two times. The three supernatants were pooled, filtered twice (paper Whatman No. 1, Maidstone, UK; Minisart™ Plus Syringe Filters, Thermo Fisher Scientific, Rodano, (MI), Italy), and the polyphenol amount and profile as well as the antioxidant activity were evaluated. The content of total polyphenols was evaluated at λ = 760 nm (Cary 50 Uv/Vis spectrophotometer, Varian-Agilent Italia, Cernusco sul Naviglio, Italy), with Folin-Ciocalteau reagent, using gallic acid as standard [22], based also on the work of Fratianni et al. [23]. The results were expressed as μg gallic acid equivalent (GAE) g^−1^ of fresh sample.

#### 2.3.2. β-Carotene Content

The extraction of the carotenoids was done by crushing 2 g of sample in 2 mL of ethanol (to allow the release the retinol); 3 mL of petroleum ether was added to it, and the mixture was vigorously shaken. Each sample was then centrifuged for 15 min at 1000× *g* (Biofuge, Beckman Italia). From the upper phase, 2 mL were taken, which was read with a spectrophotometer at 450 nm (Cary 50 MPR, Varian-Agilent Italia, Cernusco sul Naviglio, Italy), using petroleum ether as blank, and with an extinction coefficient ε = 2592. The concentration of β-carotene was established by the standard curve produced with β-carotene standard [24].

#### 2.3.3. Lycopene Content

Samples were crushed and incubated (1:5 w: vol) in a mixture composed of hexane–methanol–acetone (2:1:1 vol:vol:vol) containing 2.5% BHT (butylated-hydroxy-toluene). The supernatant was recovered and read at λ: 520 nm and λ: 450; the first wavelength was used to measure the amount of lycopene, and the second wavelength was used to cancel out the possible interferences of the other carotenoids [24]. From the difference between the two λ values, we obtained the real value of lycopene, which was calculated based on a calibration line obtained with different concentrations of pure lycopene.

#### 2.3.4. Dosage of Ascorbic Acid

The dosage of ascorbic acid was performed following the method of Nazzaro et al. [25]. Briefly, samples were homogenized (Stomacher^®^ 400 Circulator, VWR International Srl, Milano, Italy) and incubated with three volumes of metaphosphoric acid (4%) and kept for 1 h at 4 °C in the dark. Extracts were centrifuged for 10 min at 4 °C and 11,600× *g* (Biofuge, Beckman Italia, Cassina de’ Pecchi, Milano, Italy) and filtered (0.45 mm mesh, Millipore, Italy). The chromatographic determination of ascorbic acid was performed by liquid chromatography, using an RP-HPLC Gold System chromatograph with a UV detector (Beckman Italia) and a Khromasil KR 100-5 C18 column (25 cm × 4.6 mm) at room temperature. Sulphuric acid at 0.001 M in HPLC-grade water was the mobile phase used. The injection volume was 20 μL, the flow rate was set at 1.0 mL/min, and the detection wavelength was set at 245 nm. The concentration of ascorbic acid was established by the standard curve produced with ascorbic acid standard solution.

#### 2.3.5. Antioxidant Activity

The radical-scavenging activity was determined in microplates using the stable radical 2,2-diphenyl-1-picrylhydrazyl (DPPH assay) [26]. The analysis was performed by adding 15 μL of extract to 300 μL of a methanol–DPPH solution (6 × 10^−5^ M) and measuring the absorbance at λ = 517 nm (Cary 50 MPR). The absorbance of DPPH without antioxidant (control sample) was used for baseline measurements. The scavenging activity was calculated as a percentage. The experiments were performed in triplicate. Results were expressed as the mean values ± standard deviation.

#### 2.3.6. Polyphenols Chromatographic Analysis

An ACQUITY Ultra Performance LC^TM^ system (Waters, Milford, MA, USA) linked to a PDA 2996 photodiode array detector (Waters) was used for ultra-high-performance liquid chromatography (UPLC) analysis, following the method of Pane et al. [27]. The acquisition and processing of the relative data, as well as the control of the instruments was performed through the Empower software. The extracts and standards (which were previously dissolved in methanol to have different concentrations ranging from 0.001 to 5 mM) were filtered (0.45 μm; Waters, Milford, MA, USA) before analysis. The analyses were carried at 30 °C using a reversed phase column (BEH C_18_, 1.7 μm, 2.1 × 100 mm; Waters). The mobile phase consisted of solvent A (7.5 mM acetic acid) and solvent B (acetonitrile) at a flow rate of 250 μL min^‒1^. A gradient elution was employed, starting with 5% B for 0.8 min, then 5–20% B over 5.2 min, isocratic 20% B for 0.5 min, 2%30% B for 1 min, isocratic 30% B for 0.2 min, 30–50% B over 2.3 min, 50–100% B over 1 min, and isocratic 100% B for 1 min, 5–100% B over 0.5 min, and finally the column was equilibrated under the initial conditions for 2.5 min. The pressure ranged from 6000 to 8000 psi. The injection volume was 5 μL. The effluent was introduced into an LC detector (scanning range: 210–400 nm, resolution: 1.2 nm).

### 2.4. Statistical Analysis

As concerns chemical parameters, data were expressed as the mean ± the standard deviation of triplicate measurements; calculations were performed using the PC software “Excel Statistics”. Interrelationships (in terms of linear correlation) between bio-chemicals parameters and antioxidant activity of the different varieties were investigated [28] by measuring the Pearson correlation coefficients; these calculations were performed using the Matlab software package.

## 3. Results

### 3.1. Chemical Parameters

The data for some of the chemical parameters evaluated for the five landraces of the tomato “Piennolo” are shown in Table 1.

From these data, it emerged that, regarding the °Brix value, all samples exhibited a high °Brix ranging between 6.4 (“Principe Borghese”) and 7.6 (“Piennolo di Pollena”). Such values fall perfectly within the range of refractometric values provided, for example, for cherry or cluster tomatoes [29], which normally must have a °Brix between 6 and 10. A high °Brix also justifies the high shelf life of “Piennolo”. In fact, in the presence of a low °Brix, especially in the transformation and subsequent conservation processes, it is necessary to overcome this by increasing the acidity. Generally, the presence of low °Brix and acidity, as well as pH values higher than 5.0, could cause detrimental effects to the quality of the product [14,15], so that, for instance, some attempts were made to act on the genetic locus of the Brix [30] to increase the °Brix of two tomato varieties destined for processing. In the five landraces of the tomato “Piennolo”, a high °Brix was associated with a low pH, between 4.27 and 4.36. This could be indicative of the good quality of the product, as it is principally linked to the acid content of the fruit and concurs also to influence the flavor of the tomato products, which is a complex of the taste and aroma, which are affected by both physiological and chemical characteristics. These parameters are important also during the transformation processes, when an overly high pH requires the addition of acidic components [31].

The five landraces of “Piennolo”, thanks to a high °Brix, a pH lower than 5.0, and a high natural acidity, might be potentially able to be kept as a fresh product for many months even at room temperature, which is the natural status for this type of tomato, which is generally grouped and hung in a “Piennolo” style and stored outside the balconies and terraces of Naples and of many towns in Campania in general. The data obtained from the analysis could indirectly demonstrate other qualitative parameters for the five ecotypes of “Piennolo”, such as the flavor (determined by a high sugar content) and taste (determined by a not negligible total acid content, represented essentially by citric acid) [32,33].

### 3.2. Biochemical Aspects

In Table 2, we summarized the results of the analysis performed to determine the total polyphenol content, the amount of β-carotene, lycopene, ascorbic acid, and the antioxidant activity.

#### 3.2.1. Total Polyphenols

Recently, tomato polyphenols have been recognized as contributors to the modulation of several biological effects, including the ability to inhibit the in vitro acetylcholinesterase activity, which plays a key role in the symptomatic treatment of neurodegenerative diseases, including Alzheimer’s disease [34]. The five ecotypes of “Piennolo” analyzed exhibited a total polyphenol content ranging between 230 μg g^−1^ of fresh product (found in the variety “Centoscocche”) and 320 μg g^−1^ of fresh product, observed in the varieties “Piennolo di Pollena” and “Piennolo Vesuviano” (Table 2). This value was lower than that found by Carrillo et al. [15], but higher than that highlighted by Caruso et al. [19] by comparing the chemical and biochemical characteristics of the variety “Piennolo del Vesuvio” in relation to the applied cultivation methodology; furthermore, in conventional crops, a quantity of total polyphenols equal to 1.9 mg GAE in 100 g of dry product was found, even if there was undoubtedly a higher lycopene content. This confirms that the content of phenolics in tomatoes may be under genetic control [35] and may be also influenced by the environmental and climatic conditions. Previous studies have reported a high content of polyphenols in long-storage tomatoes which is potentially related to the environmental pressure, which in some cases exerted a natural selection toward those landraces abler to trigger phenol biosynthesis and accumulation [36], and as in our case, to the production of carotenoids [37].

#### 3.2.2. Carotenoids

We evaluated the amounts of two carotenoids considered important molecular parameters for the product and to be healthy key molecules, which were β-carotene and lycopene, with this last being the most important carotenoid present in tomatoes. Results are shown in Table 2. The content of lycopene ranged from 158.71 μg g^−1^ of fresh product (in the “Piennolo di Pollena”) and 218.89 μg g^−1^ of fresh product (in the “Centoscocche”). These values were higher than those found, for example, by Carrillo et al. [15] in other “Piennolo” grown in the Vesuvius town of Herculaneum, and by Fattore et al. [38], who analyzed the carotenoids through chromatographic approach and obtained a value of 78.6 mg kg^−1^ of fresh product, and found also a level of β-carotene in line with or in some cases lower than those observed in the different landraces of “Piennolo” we analyzed, which showed values of β-carotene ranging between 1.93 μg g^−1^ of fresh product (“Piennolo del Vesuvio”) and 1.068 μg g^−1^ of fresh product (“Piennolo Principe Borghese”). The other landraces had a β-carotene content corresponding to 1.34 μg g^−1^ of fresh product (“Piennolo Centoscocche”), 1.36 μg g^−1^ of fresh product (“Piennolo Rosso Vesuviano”), and 1.65 μg g^−1^ of fresh product (“Piennolo di Pollena”). The remarkable antioxidant capacity of lycopene is essentially due to its structural conformation, which allows it to release electrons and thus slow down the oxidation processes that can affect mono and polyunsaturated fatty acids [24]. This property can also extend the shelf-life of the tomato itself. It is no coincidence, in fact, that the tomato “Piennolo” is conserved by simply being exposed to the air for many months without losing—or at least losing only few—of its organoleptic and qualitative characteristics, as was also shown by Manzo et al. [39], who monitored the quantitative trends of some bioactive molecules during the 6-month storage of the product. The presence of such a high level of lycopene is of particular relevance also from a functional and health point of view. Lycopene exhibits the highest antioxidant activity and singlet oxygen quenching ability of all the dietary carotenoids [40]. As a lipid-soluble compound, the consumption of lycopene with fat increases its bioavailability. Epidemiological studies on lycopene have correlated the increase of tomato intake with a lower incidence of some pathologies, such as different types of cancer, [41,42,43,44,45]; it can contribute to fighting serum lipid peroxidation and LDL oxidation contributing also to decreasing the risk of atherosclerosis and coronary heart disease [45,46]. In recent years, lycopene has been considered an important compound with prebiotic properties and to be capable of having a positive effect on microbiomes, increasing bacterial diversity and promoting the proliferation of *Akkermansia* and also having an impact on obesity [47,48]. Thus, the fact that these landraces of “Piennolo” exhibited such a high content of lycopene potentially makes them particularly healthy food both for raw and cooked consumption, as can be done normally, compared to other types of tomatoes, such as industrial tomatoes with a much lower content of lycopene, the functional characteristics of which can only be enhanced with thermal technological processes.

#### 3.2.3. Ascorbic Acid

The ascorbic acid (AA) content ranged from 197 μg g^−1^ of fresh product (value observed in the “Piennolo di Pollena”) to 275 μg g^−1^ of fresh product (present in the “Principe Borghese”), with intermediate values of 233 μg g^−1^ of fresh product (in the “Piennolo Rosso Vesuviano”), 238 μg g^−1^ of fresh product (in the “Piennolo Centoscocche”), and 248 μg g^−1^ of product (found in the “Piennolo Vesuviano”). These values were also higher than those observed by Caruso et al. [15], who found a value of ascorbic acid value equal to 18,280 μg in 100 g of fresh product in the conventional production of Piennolo del Vesuvio, which decreased to 9780 μg in 100 g of fresh product after 6 months, confirming also the importance of ascorbic acid in preserving the organoleptic and nutritional quality of tomato. Thus, “Piennolo” could support the human body both with carotenoids and ascorbic acid, which are recognized as two of the main water-soluble tomato antioxidants present in fruit and vegetables, together with phenolic compounds. We did not monitor the trend of ascorbic acid in the varieties during the time of investigation, but considering for instance the results of Cano et al. [49], Garcia-Valverde et al. [50], and those of Del Giudice et al. [51], which reported a slight decrease of AA content during ripening, or even an increase in the last stage of ripening, the high amount of AA found in the five landraces of “Piennolo” allows to assume again its important functional role for health.

#### 3.2.4. Polyphenol Profile

The analysis of the polyphenol profile, performed by UPLC, allowed us to recognize and to quantify some metabolites. Results of the analysis are shown in Table 3.

Hyperoside, chlorogenic acid, and gallic acid resulted as the most abundant metabolites. We did not find quercetin, apigenin, or epicatechin. Among the flavonoids, we detected also very low amounts of catechin (always present although not exceeding 4 μg g^−1^ of fresh product) and a variable amount of rutin was found in almost all varieties (except “Piennolo Rosso Vesuviano”), which ranged from 6 μg g^−1^ of fresh product (“Piennolo Centoscocche”) to 34 μg g^−1^ of fresh product (discovered in “Piennolo Principe Borghese”). Some of the metabolites identified through UPLC were also identified by Del Giudice et al. [51]. In contrast to the results reported by those authors, however, we did not identify *p*-coumaric acid and naringenin; on the contrary, we found a large amount of hyperoside (quercetin 3-*O*-β-d-galactoside), which was the most abundant metabolite among those recognized by the chromatographic analysis, confirming the presence of an abundant amount of flavonoids in tomatoes. The amount of other polyphenols, such as ferulic acid and gallic acid resulted as much higher, with respect to the study carried by Vallverdu et al. [52] performed on different varieties of tomato, where the amount of ferulic and gallic acids did never exceed 0.35 and 1.20 μg g^−1^ of fresh product, respectively. The identification of ferulic acid but not of *p*-coumaric acid probably meant that the metabolic pathway did not follow the route toward the formation of lignin but took a “horizontal route”, with the subsequent production, from *p*-coumaric acid, of ferulic acid [37]. The presence of hyperoside is of remarkable importance for the nutraceutical properties of the tomato “Piennolo”, due to its anti-inflammatory effect and in the prevention of adipogenesis, obesity, and obesity-associated complications [53,54]. The concentration of some polyphenols was also higher than in other types of tomato, such as Cerise, which had a concentration of chlorogenic acid equal to 93.75 mg g^−1^ of dry product weight. The same cultivar, however, exhibited an undoubtedly much higher quantity of rutin when compared to our results [34].

#### 3.2.5. Antioxidant Activity

Tomatoes represent the main source of different biomolecules with healthy properties, including dietary lycopene. It is also an important reservoir of other important biomolecules, such as ascorbic acid and phenolic compounds, which can enhance the antioxidant activity of the tomato. Many studies have shown that the positive effect of antioxidant-rich foods on human health can be given by a pool of biomolecules that interact synergistically [10,11,50,51,52,53,54].

The antioxidant capacity and polyphenol content of tomatoes are deeply influenced by both the ripening stage and part of the fruit [39,50]; therefore, in our experiment tomato fruits were harvested at the same conditions. Furthermore, since all samples were normal-sized, the skin-volume ratio and fruit size were not expected to be a determining factor for the polyphenol content.

The landraces of “Piennolo” we analyzed exhibited an antioxidant activity ranging between 17.39% (shown by the “Piennolo Centoscocche”) and 23.91% (which was revealed by the variety “Piennolo Principe Borghese”, Table 2). The moderately high antioxidant activity exhibited by our samples could be in accordance with those reported by Ahmed et al. [54] and Odriozola-Serrano et al. [55] who, comparing the antioxidant activity of whole and cut landraces of tomato, found an antioxidant activity superior in cut than in whole products, and never exceeding 28%. Recently, Fratianni et al. [28], when evaluating the biochemical characteristics of just three types of monocultivar extra virgin olive oils, ascertained that a category of molecules can affect the antioxidant capacity of a fruit or vegetable; furthermore, within the different secondary metabolites, singular molecules can correlate more or less with the activity of a natural extract fight against different pathogens to a greater or lesser extent [56]. Carillo et al. [15] evaluated the functional characteristics of some landraces of Piennolo, including the level of total polyphenols and lycopene. Starting from these works, and taking into account that the number of tomato landraces, although superior, therefore was not high, we applied the same methodology, for the first time on these five landraces of tomato “Piennolo”, trying to evaluate what of the biochemical parameters (lycopene, β-carotene, total polyphenols and ascorbic acid, and, within polyphenols, the role of the most abundant molecules identified by UPLC) could potentially have on the antioxidant activity of the five landraces of “Piennolo”. The analysis of linear correlations suggests that the antioxidant activity was positively linked in particular to the amount of total polyphenols (ρ = 0.76, with *p* value = 0.04) and less related (or not related at all) to the content of β-carotene (ρ = 0.38) and of ascorbic acid. Lycopene, although present at a high concentration in the tomato landraces, affected in a negative way the antioxidant activity, so to give ρ = −0.96 (with *p* = 0.01). The higher influence of polyphenols on the antioxidant activity is in agreement with the findings of Ahmed et al. [54] and Patanè et al. [57]. Thus, starting from these data, tomato “Piennolo di Pollena” with the most effective antioxidant activity, displayed a high amount of total polyphenols and β-carotene, and concurrently it showed the less amount of lycopene and ascorbic acid. On the other hand, we observed that tomatoes “Piennolo Principe Borghese” and “Piennolo Vesuviano” showed similar antioxidant activity (20.41% and 20.45%, respectively, Table 2). By the analysis of the interrelationships, the value of the antioxidant activity exhibited by the “Piennolo Vesuviano” could be due to the concurrent high content of polyphenols, of lycopene, and in a minor way by β-carotene. On the other hand, the antioxidant activity of the tomato “Piennolo Principe Borghese”, could be due to the concurrent high content of total polyphenols, ascorbic acid (ρ = −0.49), and mainly by the concentration of lycopene (ρ = −0.96). Alongside, the weakest antioxidant activity found in the “Piennolo Cento Scocche” might be due to its low content of total polyphenols and β-carotene and concurrently to its high amount of lycopene. “Piennolo Rosso Vesuviano” showed a slightly higher antioxidant activity with respect to the “Piennolo Cento Scocche”, probably more due to its content of total polyphenols than related to its amount of lycopene; on the other hand, the amount of ascorbic acid and β-carotene, similar between the two landraces, were not as capable of influencing, in a different way, the antioxidant activity of the two landraces.

#### 3.2.6. Interrelationships Among the Antioxidant Activity and Singular Polyphenols

**Figure 1:**
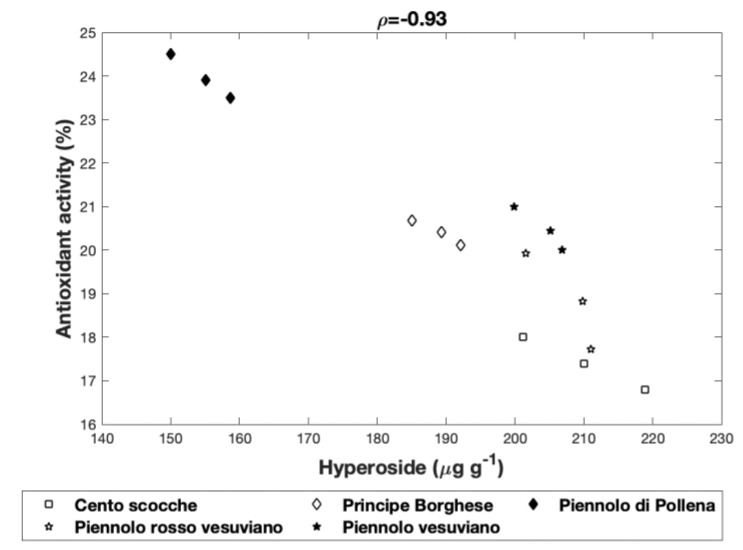
Interrelationships among the antioxidant activity exhibited by the three samples for each landrace of “Piennolo” tomato and the antioxidant activity and the content of hyperoside.

The analysis of correlation between the antioxidant activity and the most abundant metabolites, hyperoside, chlorogenic and gallic acids, identified and quantified through UPLC, revealed interesting aspects. Regarding hyperoside, the interrelationship between the concentration of this compound in each sample and the antioxidant activity indicated ρ = −0.93 (*p* = 6 × 10^−7^, Figure 1).

From these data and in disagreement with other works [58] it could be hypothesized that, although hyperoside was the most abundant molecule among the polyphenolic compounds identified by the chromatographic analysis, it did not positively affect the antioxidant activity. Therefore, following our supposition “Piennolo di Pollena” with the highest total polyphenol content and the lowest concentration of hyperoside exhibited the greatest antioxidant activity. On the contrary, tomato “Piennolo Cento Scocche”, which revealed the weakest antioxidant activity, was also characterized both by the lowest total polyphenol content and the highest hyperoside concentration. “Piennolo Rosso Vesuviano” exhibited both a total polyphenol content always higher with respect to “Piennolo Cento Scocche”, and an ever lower amount of hyperoside, which did not exceed 210 μg g^−1^; thus, hyperoside seemed to apply a slightly lower inhibiting effect on the antioxidant activity of “Piennolo Rosso Vesuviano” than on “Piennolo Cento Scocche”. “Piennolo Principe Borghese” and “Piennolo Vesuviano” showed similar antioxidant activity: in this case it could be observed that, compared to total polyphenol content, the amount of hyperoside present in the “Piennolo Vesuviano”, although superior respect to the “Piennolo Principe Borghese” resulted as proportionally inferior. Concurrently, in agreement with Slimestad et al. [59] and Apak et al. [60] chlorogenic acid and gallic acid could act in a weak manner (ρ = 0.24 and ρ = 0.18, respectively) on the antioxidant activity of the extracts of tomato landraces.

## 4. Conclusions

“Piennolo”, like other Mediterranean long storage tomatoes, constitute an important part of local heritage and culture, but it is still commercialized in local markets. Like the other tomatoes with such peculiarity, it was selected since ancient time under the typical Mediterranean climate conditions. Therefore, “Piennolo” tomato could even better respond to the varied climatic conditions, now tending to persist in higher temperatures and a lack of water resources. The high values of °Brix and acidity, as well as high sugar content and low pH, observed in the five landraces of Piennolo allowed us to consider them as products with qualitative valence, for whom it is not necessary to act modifying the chemical parameters to avoid their deterioration, so that they may be consumed both as fresh and as processed products. Furthermore, the high content of lycopene and ascorbic acid, as well as the polyphenols detected, indicate that they could represent, like other varieties of tomato [57,58,59,60,61], promising foods from a health perspective. Our results could make a contribution to knowledge about the qualitative characteristics of the systematic documentation of quality profiles of these tomato landraces, so that “Piennolo” landraces might become a valuable source of income for the local farmers, not limited to a strictly territorial marketing area.

## Figures and Tables

**Table 1:** Data of °Brix, pH value, titratable acidity (as % of citric acid), and total reducing sugars (%) present in the five landraces of tomato “Piennolo”. The analysis was performed in triplicate. In brackets the standard deviation is indicated.

Landraces	°Brix	pH	Titratable Acidity (% Citric Acid)	Total Reducing Sugars (%)
Centoscocche	6.9 (±0.1)	4.23 (±0.13)	0.56 (±0.01)	4.22 (±0.2)
Principe Borghese	6.4 (±0.12)	4.36 (±0.14)	0.58 (0.01)	4.12 (±0.12)
Piennolo di Pollena	7.6 (±0.38)	4.29 (±0.20)	0.58 (0.02)	4.37 (±0.21)
Piennolo Rosso Vesuviano	6.9 (±0.10)	4.27 (±0.20)	0.55 (±0.04)	4.72 (±0.34)
Piennolo Vesuviano	7.1 (±0.12)	4.28 (±0.15)	0.60 (±0.03)	5.19 (±0.15)

**Table 2:** Total polyphenols (TPs), β-carotene content (β-C), lycopene (Lyc), ascorbic acid content (ASA), and antioxidant activity (AA) present in the five landraces of tomato “Piennolo”. The data are shown as average of three independent experiment (±SD). Legend: PDC = Piennolo Cento Scocche; PDB = “Piennolo Principe Borghese”; PDP = “Piennolo di Pollena”; PRS = “Piennolo Rosso Vesuviano”; PDV = “Piennolo Vesuviano”.

Variety	TPs (μg g^−1^)	β-C (μg g^−1^)	Lyc (μg g^−1^)	ASA (μg g^−1^)	AA (%)
PDC	230.17 (±15.09)	1.34 (±0.023)	218.89 (±25.20)	240 (±17.0)	17.39 (±0.66)
PDB	278.12 (±13.76)	1.07 (±0.013)	192.11 (±10.99)	270 (±16.0)	20.41 (±0.27)
PDP	320.75 (±23.76)	1.65 (±0.018)	158.71 (±2.13)	190 (±2.0)	23.91 (±0.51)
PRS	296.26 (±17.03)	1.36 (±0.024)	209.84 (±14.31)	230 (±25.0)	18.82 (±1.18)
PDV	320.99 (±16.33)	1.94 (±0.02)	206.87 (±12.64)	250 (±3.0)	20.45 (±0.40)

**Table 3:** Polyphenol composition of the five landraces of tomato “Piennolo”. The data are shown as average of three independent experiments (±SD). Legend: PDC = “Piennolo Centoscocche”; PDB = “Piennolo Principe Borghese”; PDP = “Piennolo di Pollena”; PRS = “Piennolo Rosso Vesuviano”; PDV = “Piennolo Vesuviano”; CHL = chlorogenic acid; FER = ferulic acid; GAL = gallic acid; CAT = catechin; HYP = hyperoside; RUT = rutin.

Variety	CHL μg g^−1^	FER μg/g^−1^	GAL μg g^−1^	CAT μg g^−1^	HYP μg g^−1^	RUT μg g^−1^
**PDC**	98.65 (±6.12)	15.25 (±3.38)	45.22 (±5.00)	1.82 (±0.71)	210.01 (±8.88)	6.41 (±1.23)
**PDB**	125.78 (±5.01)	9.13 (±0.39)	49.97 (±5.21)	2.66 (±0.86)	188.84 (±3.53)	33.51 (±3.63)
**PDP**	128.19 (±11.97)	25.14 (±5.22)	56.82 (±4.27)	4.42 (±0.49)	154.57 (±4.39)	15.28 (±1.55)
**PRS**	146.27 (±9.53)	18.12 (±3.35)	61.35 (±4.51)	4.37 (±0.46)	207.48 (±5.16)	0.00 (±0.00)
**PDV**	154.83 (±5.30)	11.86 (±3.62)	45.14 (±3.50)	2.83 (±0.63)	203.95 (±3.65)	19.51 (±1.53)
